# Regulation of Muscle Satellite Cell Activation and Chemotaxis by Angiotensin II

**DOI:** 10.1371/journal.pone.0015212

**Published:** 2010-12-21

**Authors:** Adam P. W. Johnston, Jeff Baker, Leeann M. Bellamy, Bryon R. McKay, Michael De Lisio, Gianni Parise

**Affiliations:** 1 Department of Kinesiology, McMaster University, Hamilton, Canada; 2 Department of Medical Physics and Applied Radiation Sciences, McMaster University, Hamilton, Canada; University of Las Palmas de Gran Canaria, Spain

## Abstract

The role of angiotensin II (Ang II) in skeletal muscle is poorly understood. We report that pharmacological inhibition of Ang II signaling or ablation of the AT1a receptor significantly impaired skeletal muscle growth following myotrauma, *in vivo*, likely due to impaired satellite cell activation and chemotaxis. *In vitro* experiments demonstrated that Ang II treatment activated quiescent myoblasts as evidenced by the upregulation of myogenic regulatory factors, increased number of β-gal+, Myf5-LacZ myoblasts and the acquisition of cellular motility. Furthermore, exogenous treatment with Ang II significantly increased the chemotactic capacity of C2C12 and primary cells while AT1a^−/−^ myoblasts demonstrated a severe impairment in basal migration and were not responsive to Ang II treatment. Additionally, Ang II interacted with myoblasts in a paracrine-mediated fashion as 4 h of cyclic mechanical stimulation resulted in Ang II-induced migration of cocultured myoblasts. Ang II-induced chemotaxis appeared to be regulated by multiple mechanisms including reorganization of the actin cytoskeleton and augmentation of MMP2 activity. Collectively, these results highlight a novel role for Ang II and ACE inhibitors in the regulation of skeletal muscle growth and satellite cell function.

## Introduction

Skeletal muscle is composed of post-mitotic, multinucleated fibres. The growth, regeneration and routine maintenance (through turnover of myonuclei) is largely dependent on a population of muscle stem cells, referred to as “satellite cells”. These cells are maintained in a state of quiescence under basal conditions and become activated in response to intrinsic and environmental cues associated with muscle damage, and contraction. [1]. The activation of muscle satellite cells are characterized by the increased expression of the myogenic regulatory factors such as MyoD and Myf5 [2] and immediate early genes such as cfos [3]. Once activated, satellite cells migrate to the site of injury, proliferate and subsequently differentiate and fuse to restore skeletal muscle architecture in a process referred to as the myogenic program [4], [5]. Although much is understood about the transcriptional networks governing the myogenic program [6]–[11], little is known regarding the upstream signals or soluble factors influencing myogenic regulatory factor expression and satellite cell function.

Specifically, there is a paucity of information regarding the factors that induce the activation of satellite cells with hepatocyte growth factor being the only reliably identified cytokine [12], [13]. Similarly, the temporal kinetics, soluble factors, or signaling cascades regulating satellite cell migration are poorly understood. Indeed, chemotaxis is integral to repair and growth of skeletal muscle as satellite cells are required to migrate great distances to sites of myotrauma [14], and properly align to undergo differentiation and fusion. Interestingly, hepatocyte growth factor signaling has also been implicated in myoblast chemotaxis [15] suggesting a link between satellite cell activation and cellular motility.

Angiotensin II (Ang II) has been extensively studied in the context of its vaso-regulatory properties and the pharmacological inhibition of Ang II signaling to reduce blood pressure represents the most widely-prescribed anti-hypertensive therapy [16]. However, localized tissue renin-angiotensin systems (RAS) have been identified suggesting that Ang II may have wide ranging effects in addition to its systemic role in vasoregulation. For example, Ang II is now known to influence such diverse processes as cell proliferation, hypertrophy [17], [18] and migration [19]–[21]. Cultured skeletal muscle myoblasts and myotubes possess a local Ang II signaling system [22]; however, its function remains poorly understood. Importantly, it was reported that inhibition of Ang II signaling resulted in near complete attenuation of skeletal muscle hypertrophy in a model of synergist ablation [23], [24], suggesting that Ang II may regulate skeletal muscle hypertrophy. Regrettably, the precise role of a local RAS in skeletal muscle regeneration, growth and maintenance remains largely unknown.

The purpose of this investigation was to assess the role of Ang II in regulating the growth and repair of skeletal muscle, *in vivo*, as well as myoblast function *in vitro*. In this manuscript we report that the inhibition of Ang II signaling through captopril treatment or ablation of the Ang II type Ia receptor (AT1a) resulted in a significant impairment in skeletal muscle growth following cardiotoxin (CTX)-induced injury. Furthermore, *in vitro* experiments indicated that Ang II regulates the early satellite cell response as exogenous treatment of quiescent myoblasts with Ang II resulted in an upregulation of myogenic regulatory factor expression indicating enhanced activation, as well as an increased chemotactic capacity attributable to signaling through AT1. Ang II-induced migration occurred through reorganization of the intracellular actin cytoskeleton and enhanced matrix metalloproteinase-2 (MMP2) activity. We also report that Ang II can function in a paracrine fashion signaling neighboring myoblasts to migrate in a coculture environment. Collectively, these results identify a novel role for Ang II in the regulation of skeletal muscle growth and muscle stem cell function. Furthermore, these results suggest that the widely prescribed anti-hypertensive drug, captopril, may have adverse affects on skeletal muscle growth and repair.

## Methods

### Animals/experimental procedures

Ten-week-old C57Bl/6 mice (study-1, n = 10 per group) and fourteen-week-old AT1a^−/−^ mice and aged matched C57Bl/6 controls (study-2, n = 4 per group) (Jackson laboratories, USA) were utilized. Study-1 C57Bl/6 mice were supplemented with either normal drinking water or captopril (0.5 mg/mL, Sigma, Canada) treated drinking water three days prior to and throughout the experimental protocol. Animals were subjected to either bilateral (study-1) or unilateral (study-2) injections of CTX (25 µl at 10 µM, spread over three injections sites: proximal, mid, and distal sites of the TA) into the TA muscle and tissues were harvested 3, 10 and 21 days post injection (study-1) or 4, 7, 14 and 21 days post injection (study-2). Also, for reference of normal skeletal muscle architecture, a non-injured, non-supplemented group (n = 8) was included (study-1). Ethics approval was granted by the McMaster University Animal Research Ethics Board (AUP 06-09-51) and conformed to the guidelines of the Canadian Council on Animal Care.

### Histology

Muscles were formalin-fixed, paraffin-embedded and stained with hematoxylin and eosin (H&E) to reveal skeletal muscle architecture and viewed on a Nikon Eclipse 90i. Mean muscle fibre CSA was calculated in a blinded fashion by analyzing 300 regenerating fibres on randomly selected fields of view per animal using Nikon NIS Elements 3.0 software. β-gal activity in Myf5-LacZ cells was visualized as previously described [25]. Briefly, cells were fixed in 2 mM MgCl2 and 0.25% glutaraldehyde in PBS for 10 min followed by washing and overnight incubation in 5 mM potassium ferrocyanide, 5 mM potassium ferricyanide, 2 mM MgCl2, 4 mg/ml X-gal and 0.02% NP40 in PBS at 37°C. Cells were then washed in PBS, fixed for 10 min with 4% PFA and mounted.

### Cell culture

All cultures were maintained at 37°C in 5% CO_2_. Primary myoblasts were isolated from wild-type C57Bl/6 and AT1a^−/−^ mice as previously described [26] and cultured in primary growth media (PGM, 20% FBS in Hams F10 with 2.5 ng/mL bFGF and antibiotics). Myf5-LacZ myoblasts were a generous gift from Dr. Michael Rudnicki (Ottawa Hospital Research Institute, Ottawa Canada) and were cultured in PGM. C2C12 myoblasts were cultured in growth medium (GM, DMEM supplemented with 10% fetal bovine serum and antibiotics) or serum free medium (SFM, DMEM supplemented with antibiotics). When indicated, C2C12 and Myf5-LacZ myoblasts were rendered quiescent through 72 h of culturing in a methocellulose supplemented medium that prevented cell adhesion as described previously [27].

### Flow cytometery

The effect of Ang II treatment (24 h, 10 µM) on cell cycle kinetics of methocellulose cultured C2C12 cells was determined using PI staining or a commercially available BrdU/7AAD kit as per the manufactures instructions (cat#559619, BD pharmagen, USA) and analyzed using flow cytometry (Epics Altra, Beckman Coulter, USA).

### RNA isolation, reverse transcription and quantitative real-time polymerase chain reaction

RNA was isolated from quiescent C2C12 myoblasts (with and without Ang II (10 µM) treatment for 3 h, 6 h or 12 h) using the RNeasy method according to the manufactures instructions (Qiagen Sciences, USA) and analyzed using quantitative RT-PCR (qRT-PCR). Gene expression fold change was calculated using the delta-delta Ct method [28] using ribosomal protein L32 as a housekeeping gene. Primer sequences can be found in Table 1.

**Table 1:** Primer sequences used.

Gene	Forward primer	Reverse primer
AT1	ACAGTGATATTGGTGTTCTCAATGAAA	CCATTGTCCACCCGATGAA
cfos	GAATGGTGAAGACCGTGTCA	TGCAACGCAGACTTCTCATC
Cyclin D1	TGAACTACCTGGACCGCTTC	CCACTTGAGCTTGTTCACCA
Myf5	TGAAGGATGGACATGACGGACG	TTGTGTGCTCCGAAGGCTGCTA
MyoD	TACCCAAGGTGGAGATCCTG	CATCATGCCATCAGAGCAGT
Pax7	CTGGATGAGGGCTCAGATGT	GGTTAGCTCCTGCCTGCTTA
L32	TCCACAATGTCAAGGAGCTG	ACTCATTTTCTTCGCTGCGT

### Transwell/invasion/checkerboard assays

Transwell assays were conducted using proliferating or quiescent C2C12 myoblasts and primary or AT1a^−/−^ myoblasts as described previously [29] with minor modifications using 6 or 24 well, 8 µm pore transwell systems. Cells were allowed to migrate for 12 h with the lower chamber of the well containing GM, 20% FBS in DMEM, PGM or SFM with or without Ang II (10 µM) or an MMP2 inhibitor (1 µM; cat# 44424, Calbiochem, USA) where indicated. Invasion and checkerboard assays were performed in the same fashion as migration assays with the exception that transwells were pre-coated with 1% gelatin or media on either the top or the bottom of the transwell was supplemented with Ang II (10 µM). Migration was assessed by staining the cells with crystal violet, removing the cells on the upper side of the transwell and counting the number of migrated cells in 15 random fields of view at 40x magnification using Zeiss Axiovert 200 microscope (Carl Zeiss Canada Ltd.) or by solubilizing the cells in 1% triton X-100 and measuring the absorbance of the triton X-100 solution using an Ultraspec 3000 Pro (GE Healthcare, USA) at 595 nm.

### Under agarose (UA) migration assay

To further assess the chemotactic capacity of C2C12 cells in response to Ang II treatment, an under agarose migration assay [30] was performed as depicted in Figure S3. 1% agarose was polymerized in 10% FBS in DMEM in 35 mm culture plates and 3 wells were cut. C2C12 cells were added to the centre well while GM with or without Ang II (100 µM) was added to the outer wells and allowed to incubate for 14 h. The total number of cells and distance migrated under the agarose was analyzed using a Zeiss Axiovert 200 microscope (Carl Zeiss Canada Ltd). Maximal migration distance was calculated by averaging the distance of the top 15 migrating cells per sample.

### Immunohistochemistry

C2C12 cells were plated into the centre well of an under agarose migration plate while GM with or without Ang II (100 µM) was then added to one of the outer wells and incubated for 6 h. Cells were fixed in 4% PFA for 10 min (or 5 min, followed by dehydration in ethanol for co-staining). IHC staining for AT1 receptor was done using anti-AT1 (cat# SC-1173, Santa Cruz, USA) and revealed with anti-rabbit Alexa 488 secondary antibody (Molecular Probes, USA). Filamentous actin was visualized with TRITC-conjugated phalloidin (0.2 µg/mL, cat#P1951, Sigma-Aldrich, Canada) and incubated simultaneously overnight at 4°C with anti-AT1 for costaining and counterstained with DAPI.

### Mechanical stretch/coculture migration assay

Flexible bottom culture plates (Flexcell International, USA) were modified to accommodate a transwell insert. C2C12 myoblasts were pretreated for 36 h in either GM or GM supplemented with captopril (10 mM) to inhibit endogenous Ang II production. 2×10^5^ cells were seeded onto type I collagen coated flex-cell plates while 5×10^4^ cells were seeded into the upper well of a transwell insert and placed into the flex-cell plates. Cells were subjected to a 4 h cyclic strain protocol (of 0.1 Hz at 20% strain) applied using the FX-4000 Tension Plus (Flexcell International, USA) in the presence or absence of captopril. After 20 h, cell migration was assessed as described above.

### Gelatin Zymography

C2C12 myoblasts were cultured in GM and treated with Ang II (10 µM) for 48 h and media and total protein was collected analyzed using gelatin zymography as previously described [31]. 20 µl of media or 7.5 µg of protein was loaded into a 10% polyacrylamide gel containing 1% gelatin and resolved for 90 min at 100 V. Gels were incubated in 2.5% triton X-100 for 1 h, washed in H_2_O for 2×20 min, and incubated for 20 h at 37°C in a 50 mM Tris-HCl buffer, pH 8.0, containing 5 mM calcium chloride. Gels were then fixed with 50% methanol, 10% acetic acid containing 0.25% Coomassie Blue. Bands appear clear against the stained gel and were visualized using an Alpha Innotech FluroChem SP and quantified using Alphaease software.

### Statistical analysis

Analysis of *in vivo* measures was done by two-way ANOVA. In vitro experiments were analyzed using t-tests and one-way ANOVA where appropriate. T-tests were also performed on gene expression fold change for qRT-PCR analysis.

## Results

### Ang II signaling mediates skeletal muscle growth following CTX injury

**Figure 1:**
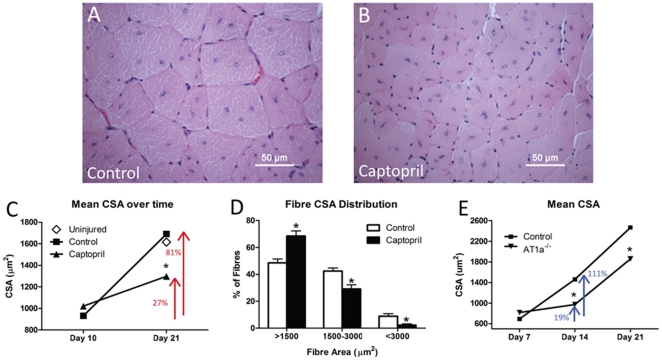
Inhibition of Ang II signaling abrogates skeletal muscle growth following CTX injury. Representative H&E stains of TA cross-sections of (A) control and (B) captopril treated mice 21 days after CTX injection (both photos - 40x magnification). C) Mean muscle fibre CSA of control and captopril treated mice 10 and 21 days following CTX injection. Note: ◊ - indicates CSA of uninjured mice (n = 6 per group). D) Distribution of small (<1500 µm^2^), medium (1500–3000 µm^2^) and large (>3000 µm^2^) size muscle fibres of control and captopril treated mice 21 days following CTX injection. E) Analysis of muscle fibre CSA of control and AT1a^−/−^ mice 7, 14 and 21 days following CTX injection (n = 4 per group). Data are presented as mean ± s.e.m, *indicates a significant difference (p<0.05) from control.

To determine the *in vivo* consequence of impaired Ang II signaling on muscle regeneration and growth we injected AT1a^−/−^ or ACE inhibitor (captopril) treated mice with CTX to induce myotrauma. Analysis of H&E stained TA cross-sections revealed that in comparison to controls, captopril supplemented mice presented with a decrease in muscle fibre cross-sectional area (CSA) of ∼25% 21 days following injury (Figure 1A, B, C; p<0.05) while no differences were observed between groups 10 days post injection. 21 days also appeared to be sufficient time for control animals to fully regenerate as fibre CSA was not different from uninjured (UI) mice while captopril treated mice still demonstrated a significant ∼25% reduction in CSA relative to UI mice (Figure 1C). Importantly, CTX-induced damage was evident in over 90% of muscle fibres in both groups as represented by the presence of central nuclei in almost all fibres 21d post injury, and the fibre CSA differences between groups was overt and evident in 10X images (Figure S6). Analysis of small (<1500 µm), medium (1500 to 3000 µm) and large (>3000 µm) size fibres revealed an increased frequency of small fibres and a decrease in large fibres with captopril treatment (Figure 1D). Furthermore, when muscle fibre CSA was analyzed from days 10–21 post injury we observed that captopril treatment significantly impaired muscle growth as control animals demonstrated an 81% increase in CSA while captopril treated mice increased by only 27% (Figure 1C). To investigate the role of Ang II receptor sub-type we repeated similar experiments inducing regeneration in AT1a^−/−^ mice. In agreement with the captopril treated animals, AT1a^−/−^ mice also revealed impaired skeletal muscle growth following the formation of myofibres with significant decreases in fibre CSA of ∼35% and ∼25% at 14d and 21d respectively compared to controls (Figure 1E). When the rate of growth was determined between day 7–14 post injury, a 110% increase in fiber CSA was documented in the WT mice while a non-significant 19% increase was observed in AT1a^−/−^ mice (Figure 1E). Importantly, normal skeletal muscle growth was not impaired in AT1a^−/−^ mice as no differences were observed in myofibre CSA between uninjured WT and AT1a^−/−^ mice (Figure S7), however our data suggests that post-natal induced muscle growth following injury requires Ang II.

### Ang II activates quiescent myoblasts without enhancing proliferation

**Figure 2:**
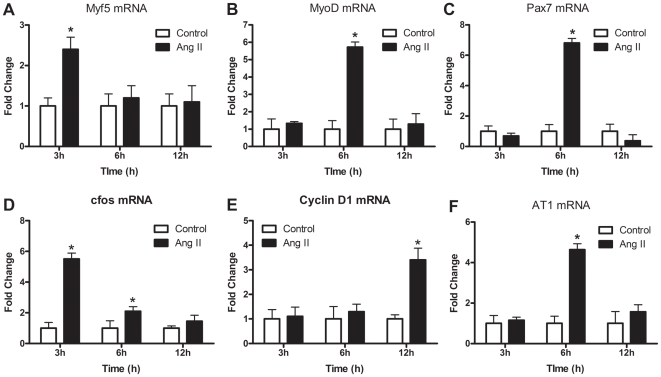
Ang II treatment upregulates mRNAs found within activated satellite cells. qRT-PCR analysis of (A) Myf5, (B) MyoD, (C) Pax7, (D) cfos, (E) cyclin D1, and (F) AT1 in quiescent C2C12 myoblasts in response to Ang II treatment for 3, 6 or 12 h (n = 6 per group). Data are presented as mean ± s.e.m, *indicates a significant difference (p<0.05) from control.

**Figure 3:**
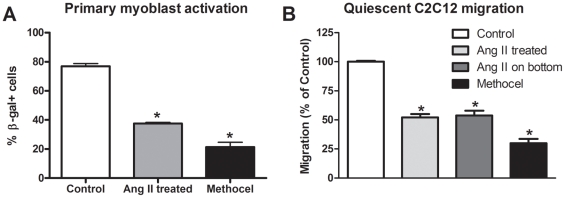
Ang II treatment results in the activation of primary myoblasts and the acquisition of motility. A) Actively dividing Myf5-LacZ myoblasts (control) were cultured in PGM while quiescent Myf5-LacZ myoblasts were treated with Hams F10 (methocel) or Ang II for 6 h and the percentage of β-gal+ cells was assessed (n = 6 per group). B) Quiescent C2C12 myoblasts were treated with SFM (methocel), Ang II directly (Ang II treatment), an Ang II concentration gradient (Ang II on bottom) or 20% FBS (control) and migration was measured using transwells (n = 6 per group). Data are presented as mean ± s.e.m, *indicates a significant difference (p<0.05) from control.

To investigate the mechanisms responsible for repressed muscle growth, due to inhibition of Ang II signaling, we analyzed the effect of Ang II treatment on myoblast function. The use of methocellulose culture suspension to induce quiescence has previously been validated and is a valuable tool to assess myoblast activation [32]–[34]. To confirm quiescence of methocellulose treated cells we analyzed the cell cycle kinetics using propidium iodide staining and flow cytometry following 72 h of methocellulose treatment. When compared to actively dividing C2C12 myoblasts, 72 h of methocellulose suspension induced synchronization of cells in G1, reduced the number of actively dividing cells (S-phase cells) to less than 1.5% (Figure S1) and decreased the number of cells progressing through the cell cycle (28% in controls vs. 11% in methocellulose treated, (Figure S1)). To assess if Ang II could activate myoblasts, quiescent C2C12 cells were treated with Ang II for 3, 6 or 12 h. Treatment of quiescent cells with Ang II induced the upregulation of mRNA species characteristic of activated myoblasts such as increases in the myogenic regulatory factor Myf5 (2.5-fold) and MyoD (6-fold) at 3 h and 6 h respectively as well as Pax7 (7-fold, 6 h) (Figure 2A, B, C). Ang II treatment also led to a significant increase in the cell cycle gene cyclin D1 as well as an early (3 h) increase in cfos gene expression (Figure 2D, E), which has been proposed to be one of the earliest events associated with satellite cell activation *in vivo* [3]. Additionally, Ang II treatment increased the expression of the AT1 receptor in quiescent myoblasts as a 5-fold increase was observed following 6 h of treatment (Figure 2F). Since satellite cell activation is characterized by an upregulation of Myf5 [5] and to confirm that Ang II activated quiescent primary myoblasts, the effect of 6 h of Ang II treatment was assessed in Myf5-LacZ primary myoblasts following 72 h of methocellulose culture suspension. In comparison to actively dividing cells, methocellolose culture reduced the number of β-galactosidase (β-gal)+ cells from ∼77% to ∼21% (Figure 3A; p<0.05) confirming the quiescence of these cells. However, Ang II treatment of quiescent myoblasts successfully increased the number of β-gal+ cells by ∼75% (Figure 3A; p<0.05).

Next, we wanted to assess whether the activation of quiescent myoblasts induced cell motility. Therefore, we analyzed the migratory capacity of quiescent myoblasts treated with serum free media (methocel), Ang II or 20% FBS supplemented media (control), which is a known inducer of myoblast activation. When compared to controls, cells treated with SFM displayed a severe impairment of migratory capacity (∼70% reduction, Figure 3B) confirming that quiescent cells must become activated in order to undergo migration. Interestingly, when comparing the migratory activity of cells treated with SFM or Ang II, we demonstrate that both direct treatment and a concentration gradient of Ang II significantly increased the chemokinetic migration of cells by ∼75% (Figure 3B) demonstrating a functional measure of myoblast activation.

Since we demonstrated that Ang II activates quiescent cells and upregulates key transcription factors necessary for myoblast proliferation (i.e. Myf5, cyclin D1), we tested whether Ang II possessed the ability to induce quiescent cells to proliferate. Interestingly, no differences were observed between groups in either the number of BrdU positive cells or the cell cycle kinetics in response to 24 h of Ang II treatment of quiescent myoblasts (Figure S2). Additionally, 6 h of BrdU incorporation into primary myoblasts treated with Ang II was not different from cells treated with control media providing further evidence that Ang II alone does not play a role in cell proliferation (Figure S8). Collectively, these results demonstrate that Ang II activates myoblasts and initiates cell cycle entry but does not directly induce proliferation of quiescent myoblasts.

### Ang II signals through the AT1a receptor to induce chemotaxis of proliferating cells

**Figure 4:**
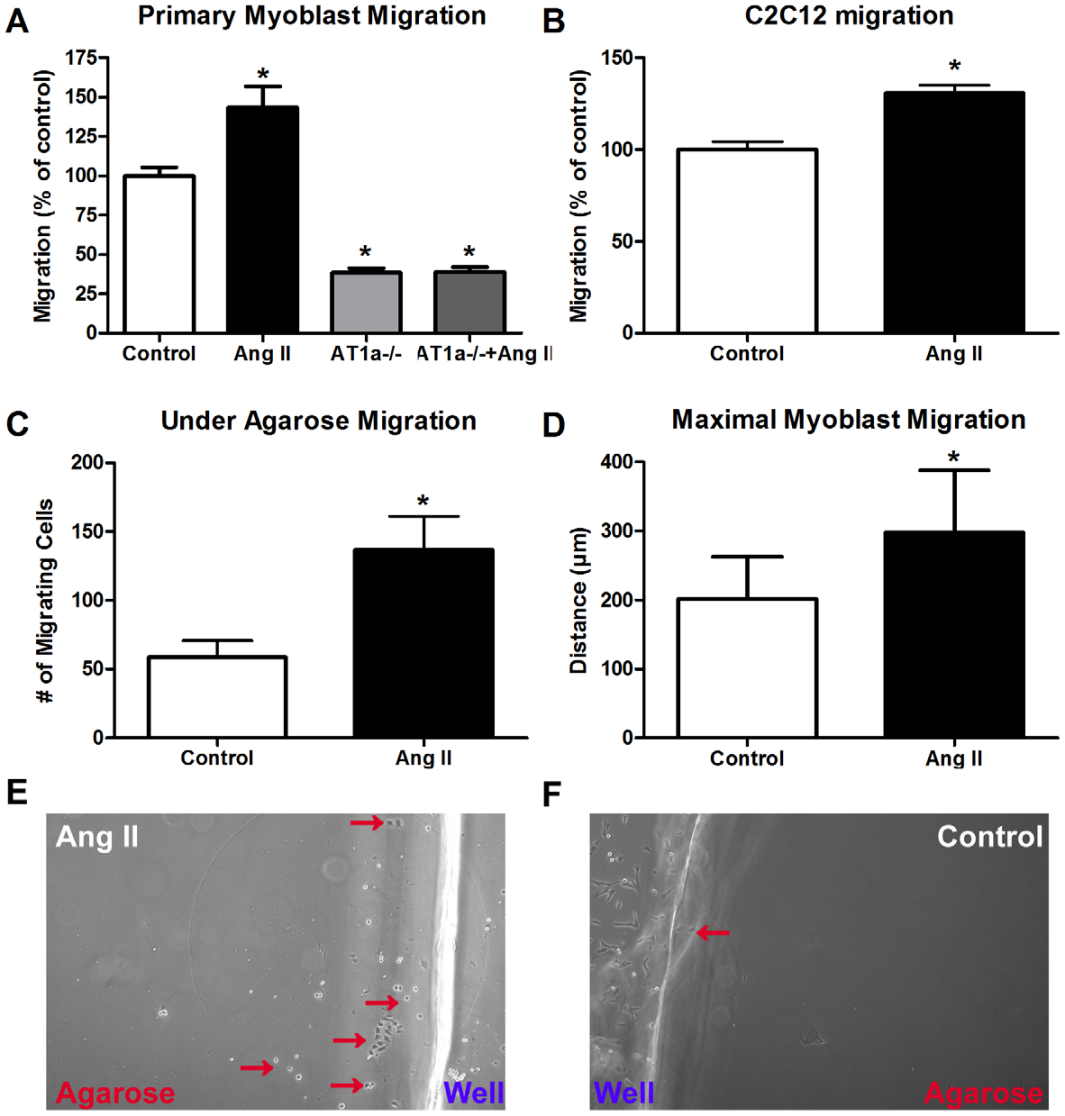
Ang II signals through the AT1a receptor to increase myoblast chemotaxis. Analysis of (A) primary WT, AT1a^−/−^ and (B) C2C12 myoblast migration through transwells in response to Ang II treatment (n = 10 per group). Analysis of (C) total number and (D) maximal distance of C2C12 myoblasts following 12 h of UA migration (n = 16 per group). Representative images of Ang II treated (E) and control (F) C2C12 cells following 12 h of UA migration. *indicates a significant difference (p<0.05) from control.

Since the process of myoblast chemotaxis is poorly understood and Ang II has been implicated in the motility of several cell types [19]–[21], we assessed the role of Ang II in regulating myoblast chemotaxis using an assay commonly utilized to assess inflammatory cell migration [30]. Under agarose migration analysis (Figure S3) revealed a robust 133% increase in the number of C2C12 cells that migrated out of the centre well in response to an Ang II concentration gradient (Figure 4C) as well as increasing the maximal distance migrated by ∼50% (Ang II-298.3 µm vs. Con-201.7 µm p<0.05; Figure 4D). This increase in C2C12 migration was also evident using transwell assays where Ang II treatment induced a 30% increase in migratory capacity (Figure 4B). There are two distinct forms of myoblast motility: 1. chemokinesis, analogous to stochastic movements, and 2. chemotaxis, analogous to directed homing. Checkerboard assays revealed that when activated myoblasts were treated directly with Ang II no increase in migration was observed; however, when exposed to a concentration gradient of Ang II, a significant increase in the number of migrating cells was demonstrated (Figure S4A). To confirm that Ang II augments primary myoblast migration and to delineate which receptor subtype was activated during Ang II-induced myoblast chemotaxis, primary myoblasts were harvested from C57Bl/6 (wild-type; WT) and AT1a^−/−^ mice. Transwell assays revealed that exogenous Ang II treatment significantly increased primary myoblast chemotaxis by 43% while AT1a^−/−^ myoblasts demonstrated a profound (∼62%) inhibition (p<0.05) of migratory capacity and did not respond to Ang II treatment (Figure 4A).

**Figure 5:**
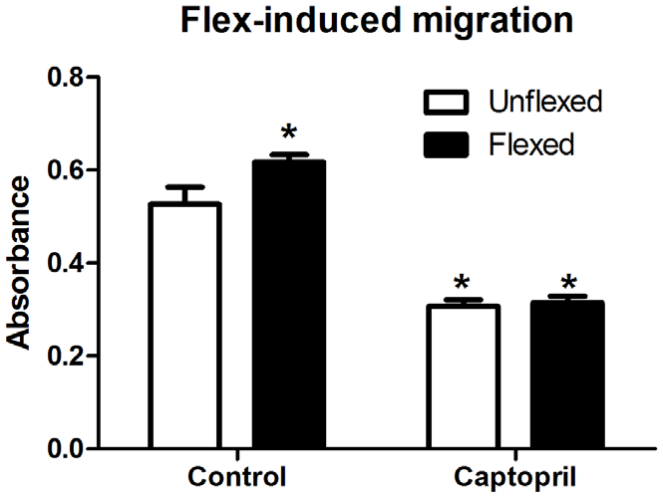
Mechanical stretch stimulates Ang II-mediated chemotaxis in coculture. Analysis of C2C12 chemotaxis in response to coculture with myoblasts exposed to 4 h of cyclic mechanical stretch in the absence or presence of captopril (n = 6 per group). *indicates a significant difference (p<0.05) from control-unflexed. All data are presented as mean ± s.e.m.

We have previously demonstrated that myoblasts locally produce Ang II and express a “stretch-responsive” local angiotensin signaling system [22]. Based on these findings, we explored the possibility that the mechanical stretch of myoblasts could induce the production of Ang II, which in turn, could initiate the migration of cocultured myoblasts. Our results demonstrate a significant 17% increase in myoblast migration in response to factors released from myoblasts undergoing stretch (Figure 5A) that was abolished when myoblasts underwent mechanical stretch in the presence of captopril (Figure 5B). Moreover, captopril treatment of unflexed cells also significantly inhibited the basal migration of myoblasts (p<0.05). Collectively, these data indicate that Ang II signaling through the AT1a receptor is a mediator of myoblast chemotaxis and that Ang II can signal chemotaxis in a paracrine fashion.

### Ang II-induced chemotaxis is mediated by multiple mechanisms including cytoskeletal reorganization and increased MMP2

**Figure 6:**
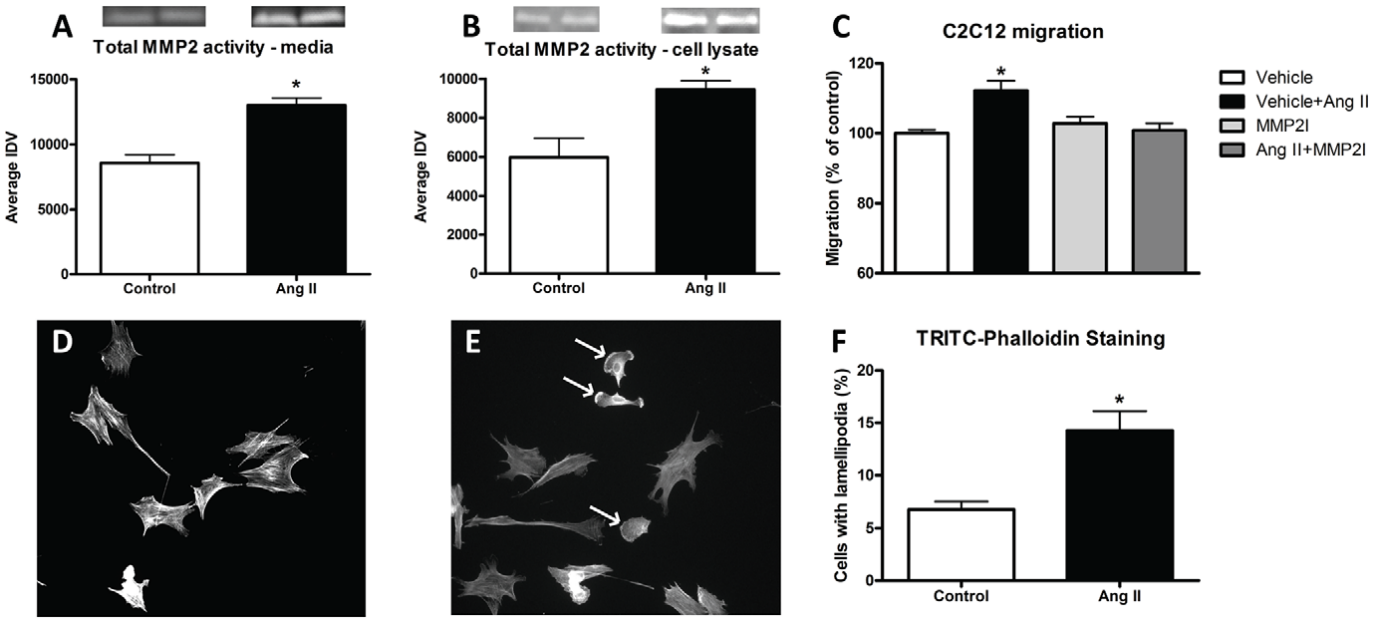
Ang II treatment regulates myoblast chemotaxis through increased MMP2 and actin cytoskeletal reorganization. A) Gelatin zymography analysis of total MMP2 activity within cell culture media and (B) cell lysates of C2C12 myoblasts treated with Ang II (n = 6 per group). C) Analysis of the migration of C2C12 myoblasts treated with Vehicle, Vehicle+Ang II, MMP2 inhibitor or Ang II+MMP2 inhibitor (n = 6 per group). F) Analysis of TRITC-conjugated phalloidin staining of (D) control and (E) Ang II treated C2C12 myoblasts (20x magnification, n = 12 per group). Note: arrows indicate cells displaying lamellipodial projections. Data are presented as mean ± s.e.m, *indicates a significant difference (p<0.05) from control.

Since proper actin filament assembly has been hypothesized to be a prerequisite for directed cellular motility [35], and implicated in Ang II-induced migration of other cell types [36], [37], we treated C2C12 myoblasts with a concentration gradient of Ang II and assessed the number of cells displaying altered cytoskeletal characteristics. TRITC-conjugated phalloidin staining of the actin cytoskeleton revealed that Ang II treatment increased the number of cells displaying lamellipodial projections by 111% (Figure 6D–F). Interestingly, IHC costaining of AT1 and phalloidin revealed that AT1 translocates to the leading edge of the cell and was concentrated in lamellipodia (Figure S5). These results indicate that Ang II signaling induced cellular polarization and reorganization of the actin cytoskeleton. Another important component of cell migration is the ability of a cell to degrade its extracellular environment to promote cell motility. Therefore, we assessed whether Ang II influenced enzyme activity involved in the breakdown of the extracellular matrix (ECM). Gelatin zymography analysis demonstrated an ∼40% increase in total MMP2 activity in both the cell culture media and cell lysate (Figure 6A, B) of C2C12 cells treated with Ang II. These results are in agreement with *in vitro* invasion assays, which demonstrated that Ang II treatment of myoblasts induced a significant increase in the number of cells invading the gelatin coated transwells (Figure S4B), presumably by inducing the enzymes involved in the degradation of extracellular matrix proteins. To confirm the importance of Ang II-induced MMP2 activity in myoblast migration, a transwell assay was conducted in the presence of an MMP2 inhibitor (MMP2I). Results revealed that inhibition of MMP2 did not appear to affect basal migration of C2C12 cells, however, when cells were treated with Ang II and the MMP2I, the observed increase in migratory capacity was completely abolished (Figure 6C). These data suggest that Ang II-stimulated chemotaxis was regulated, at least in part, by MMP2 activity.

## Discussion

The regeneration and growth of skeletal muscle is largely dependent on the capacity of muscle satellite cells to efficiently respond to directed cues that signal the activation, motility and progression of satellite cells through the myogenic program. An impairment of any of these processes could result in the decreased capacity for repair or growth. Consequently, we have identified Ang II as a novel regulator of muscle stem cell chemotaxis, an activator of quiescent myoblasts and a necessary factor for induced myofibre growth following injury.

In recent years, Ang II has been described as having wide ranging biological effects independent of its vasoactivity [38]. The function of Ang II signaling in skeletal muscle and associated muscle satellite cells remains incompletely described with the potential to influence muscle hypertrophy [23], [24]. Our *in vivo* observations support a regulatory role for Ang II in mediating muscle fibre growth likely through muscle satellite cell activation and chemotaxis. Specifically, we demonstrate that both captopril treatment and ablation of the AT1a receptor resulted in a similar inhibition of fibre growth highlighting a role for AT1 mediated signaling. Importantly, muscle fibre CSA was not different between uninjured control and AT1a^−/−^ mice. This indicates that Ang II likely does not influence embryonic or neonatal muscle development but appears to only influence postnatal myogenesis during regeneration and growth.

To explain the observed inhibition of skeletal muscle growth due to captopril treatment, we focused our analysis on the potential of Ang II to mediate the early response of muscle stem cells to muscle repair since very little is known regarding the factors that mediate this phase. We demonstrate that Ang II induced the expression of mRNAs and protein known to be upregulated in activated myoblasts such as the myogenic regulatory factors Myf5, MyoD and Pax7. Interestingly, the temporal expression of these genes in response to Ang II treatment is in agreement with *in vivo* data demonstrating that satellite cell activation is represented by an early upregulation of Myf5 followed by increased MyoD expression [39]. Importantly, Ang II treatment lead to the production of functional Myf5 protein in cultured primary cells proving that effects of Ang II on myoblast activation are not restricted to C2C12 cells. We also demonstrated that the activation of myoblasts resulted in acquired cellular motility, which likely serves to upregulate the migratory machinery necessary to respond to chemotactic signals. It is interesting to note that Ang II treatment of quiescent cells induced the same magnitude increase in chemokinesis of C2C12 cells as that observed in the number of β-gal+ Myf5-LacZ cells, further supporting the relationship between Ang II induced activation and cellular motility. These results suggest that early responses of satellite cells to myotrauma may be coordinated by Ang II as it plays a pleiotropic role in activating cells as well directing their subsequent chemotactic response.

Our data also demonstrate that although Ang II activated myoblasts, it did not alter cell cycle kinetics of quiescent cells. These data suggest that Ang II primarily functions to activate myoblasts but may act in concert with other factors to induce full cell cycle entry and proliferation. This theory is supported by Hlaing and colleagues [40] who demonstrated that Ang II treatment of serum starved C2C12 cells had no effect on myoblast proliferation but induced the transient activation of Cdk4, Rb phosphorylation and the subsequent release of HDAC1. However, they also reported that although Ang II transcriptionally activated E2F-1, this complex did not dissociate from the Rb protein subsequently suppressing genes necessary for cell cycle progression.

The chemotaxis of muscle stem cells to the site of injury is an ill defined, necessary component of the early response of satellite cells to myotrauma. Using several techniques, the present experiments demonstrate that exogenous Ang II treatment significantly increased both primary and C2C12 chemotaxis. We have previously reported that myoblasts have the ability to secrete Ang II and that mechanical stimulation of C2C12 myoblasts resulted in the upregulation of RAS family member gene expression [22]. Here, we demonstrate that mechanical stretch of cells induced the chemotaxis of cocultured myoblasts through Ang II mediated mechanism(s). Although the magnitude of increase in migration was less than that induced by Ang II treatment, the concentration of Ang II released into the culture media was likely magnitudes lower, highlighting the robustness of the effect of Ang II on motility. These results suggest that mechanical stimulation due to muscle contraction *in vivo* may stimulate Ang II release that functions to recruit muscle satellite cells to the site of injury. Ang II treatment of both quiescent and proliferating cells resulted in enhanced cellular motility with quiescent cells responding to Ang II treatment chemokinetically and proliferating cells undergoing chemotaxis. Based on our results, we propose that Ang II functions to activate quiescent myoblasts subsequently increasing their motility but then serves to direct the homing response following their activation.

The source of Ang II during muscle regeneration or the molecular mechanisms regulating its production remain unknown. We have previously demonstrated that muscle cells express a local Ang II signaling system with the ability to secrete Ang II into the culture media. Therefore, regenerating muscle fibres could potentially signal the activation and chemotaxis of satellite cells to the site of injury through increased Ang II release. Furthermore, based on our coculture experiments, the myoblasts themselves may increase Ang II secretion to direct the regenerative response following myotrauma. It is important to note that angiotensin II release from damage skeletal muscle tissue, in vivo, has not yet been quantified and remains a significant priority. Of particular importance is the acknowledgement that the source of Ang II could also be a result of a local RAS within the muscle vasculature, the inflammatory response associated with muscle damage, arrive through systemic sources [38], or any combination of these scenarios. Therefore, further investigation into the contribution and localization of Ang II secretion in response to muscle injury is necessary.

Chemotaxis can be best viewed as a cyclical process involving cellular polarization, extension, contraction and detachment [35], [41], [42]. Interestingly, Ang II appeared to enhance numerous aspects of this process. Integral to the initiation of chemotaxis is the formation of actin-rich lamellipodia that serve to directionally extend the migrating cell forward [43]. The present results demonstrate that Ang II treatment induced cytoskeletal reorganization and increased the number of cells possessing lamellipodia consistent with other reports demonstrating actin cytoskeletal rearrangement and lamellipodial formation in Ang II induced migration [36], [37]. MMP2 is a gelatinase that is known to regulate migration by facilitating detachment from the ECM as well as increasing the space for cellular expansion as the cell migrates toward the degraded matrix [41]. We chose to focus our attention on MMP2 for two reasons. Firstly, this is the primary MMP expressed in myoblasts [44] and secondly, we demonstrate that Ang II increased myoblast invasion through gelatin covered transwells. In agreement with the current study, El Fahime and colleagues [44] reported that pharmacological inhibition of MMPs severely inhibited *in vivo* migration of transplanted C2C12 myoblasts while overexpression of MMP2 significantly increased their *in vivo* migratory capacity.

Ang II possesses the capacity to bind to two distinct receptor subtypes, AT1 and AT2 [45]. We have previously demonstrated that both C2C12 and primary myoblasts express both of these receptor subtypes and therefore either could regulate Ang II mediated migration. Our results highlight the role of AT1a signaling as AT1a^−/−^ primary myoblasts were severely impaired (62% reduction compared to controls) in their basal migratory capacity and did not respond to exogenous Ang II treatment. Interestingly, IHC staining of the AT1 receptor revealed its localization to lamellipodial projections in myoblasts. The functional significance of this relationship is currently unknown, however, the G-coupled chemokine receptors CCR2 and CCR5 redistribute to the leading edge of the cell during chemotaxis in lymphocytes (Nieto et al. 1997) and natural killer cells [46]. Similarly, the receptor for urokinase-type plasminogen activator undergoes translocalization to the leading edge of migrating human monocytes [47]. Therefore, the localization of AT1 at the leading edge may promote site directed accumulation of pro-migratory signalling molecules.

Collectively, our findings identify Ang II as a novel regulator of skeletal muscle growth through modulating muscle satellite cell activation and migration. Clinically, ACE inhibitors and angiotensin receptor blockers are amongst the most commonly prescribed medications [16] with >65 million individuals in the United States considered clinically hypertensive with the highest prevalence in the elderly [48], [49]. Unfortunately this population is also at the highest risk of muscle loss due to age. Interestingly, a recent observational study by Onder and colleagues [50] demonstrated that chronic ACE inhibitor treatment slowed the age-related decline in muscle strength in elderly, hypertensive women. Although the mechanisms underlying this observation are not understood, these seemingly conflicting findings punctuate the importance of understanding the role of Ang II in skeletal muscle and highlights the need for further investigation into the effects of Ang II signaling inhibition in elderly individuals undergoing pharmacological blockade of Ang II signaling.

## Supporting Information

Figure S1 **Methocellulose culturing synchronizes C2C12 cells in G1.** Representative cell cycle profile of flow cytometry analysis of PI stained C2C12 cells cultured in (A) GM or (B) 1.5% methocellulose for 72 h (n = 5 per group).
(TIF)

Figure S2 **Ang II treatment does not induce proliferation or alter cell cycle kinetics of C2C12 myoblasts.** Representative flow cytometery profiles of BrdU staining of (A) quiescent control and (B) Ang II treated C2C12 cells (n = 6 per group). Representative cell cycle profiles of 7AAD staining in (C) control and (D) Ang II treated C2C12 cells (n = 6 per group).
(TIF)

Figure S3 **Depiction of the under agarose migration assay.**
(TIF)

Figure S4 **Ang II treatment increases myoblast chemotaxis and invasion.** A) Analysis C2C12 myoblasts either directly treated (Ang II on top) or subjected to a concentration gradient (Ang II on bottom) of Ang II. B) Analysis of the capacity of control and Ang II treated C2C12 cells to invade gelatin coated transwells (n = 6 per group). Data are presented as mean ± s.e.m *indicates a significant difference (p<0.05) from control.
(TIF)

Figure S5 **AT1 colocalizes with lamellipodial projections.** IHC staining of (A) DAPI, (B) AT1, (C) phalloidin and (D) merge in C2C12 myoblasts (100x magnification). Arrows indicate colocalization of AT1 to lamellipodial projections of a polarized cell.
(TIF)

Figure S6 **H&E stain of TA sections at 21d of regeneration.** H&E stains at low magnification (10X) demonstrate that 1) all fibres contain central nuclei demonstrating homogeneity of injury, and 2) fibres from captopril treated animals were obviously smaller.
(TIF)

Figure S7 **Comparison of AT1a^−/−^ and control uninjured CSA.** Representative sections of uninjured TA muscles from control and AT1a^−/−^ mice demonstrate no difference in mean fibre CSA.
(TIF)

Figure S8 **BrdU incorporation into freshly isolated satellite cells treated with Ang II.** Additional evidence demonstrating that Ang II does not appear to influence cell proliferation.
(TIF)
