# Supplementary figures and images for: Regulation of Muscle Satellite Cell Activation and Chemotaxis by Angiotensin II
Source: PLoS One. 2010 Dec 21;5(12):e15212. doi: 10.1371/journal.pone.0015212 (PMC3006204; doi:10.1371/journal.pone.0015212)

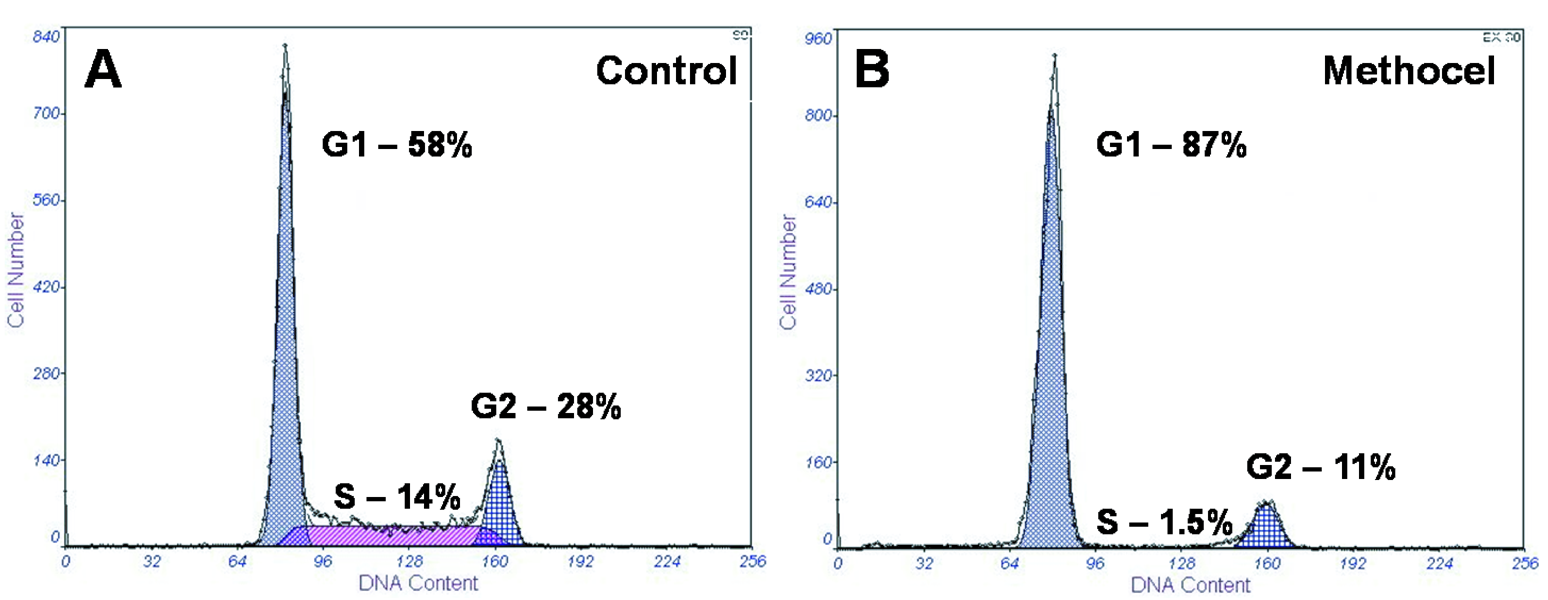

Supplement: Figure S1 — Methocellulose culturing synchronizes C2C12 cells in G1. Representative cell cycle profile of flow cytometry analysis of PI stained C2C12 cells cultured in (A) GM or (B) 1.5% methocellulose for 72 h (n = 5 per group). (TIF) [file pone.0015212.s001.tif]

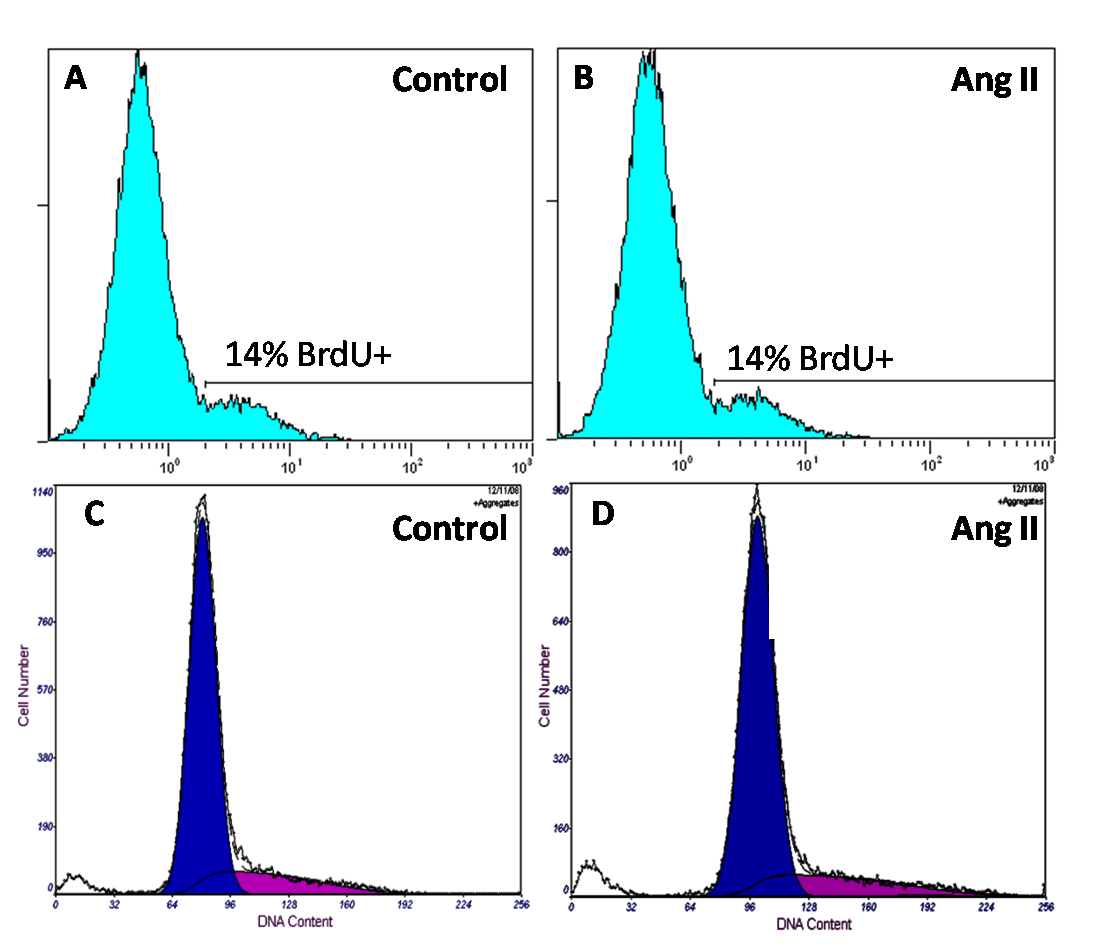

Supplement: Figure S2 — Ang II treatment does not induce proliferation or alter cell cycle kinetics of C2C12 myoblasts. Representative flow cytometery profiles of BrdU staining of (A) quiescent control and (B) Ang II treated C2C12 cells (n = 6 per group). Representative cell cycle profiles of 7AAD staining in (C) control and (D) Ang II treated C2C12 cells (n = 6 per group). (TIF) [file pone.0015212.s002.tif]

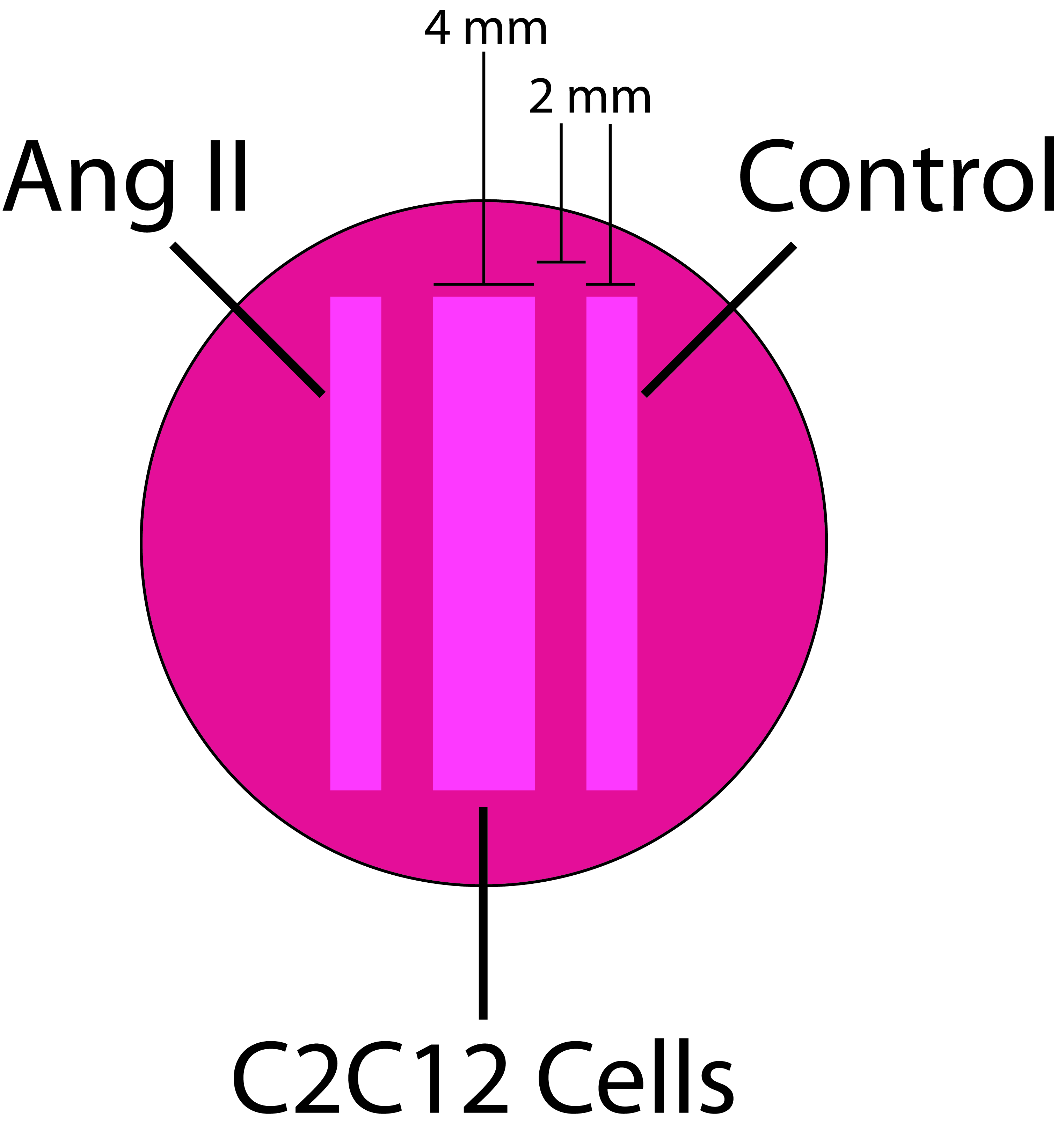

Supplement: Figure S3 — Depiction of the under agarose migration assay. (TIF) [file pone.0015212.s003.tif]

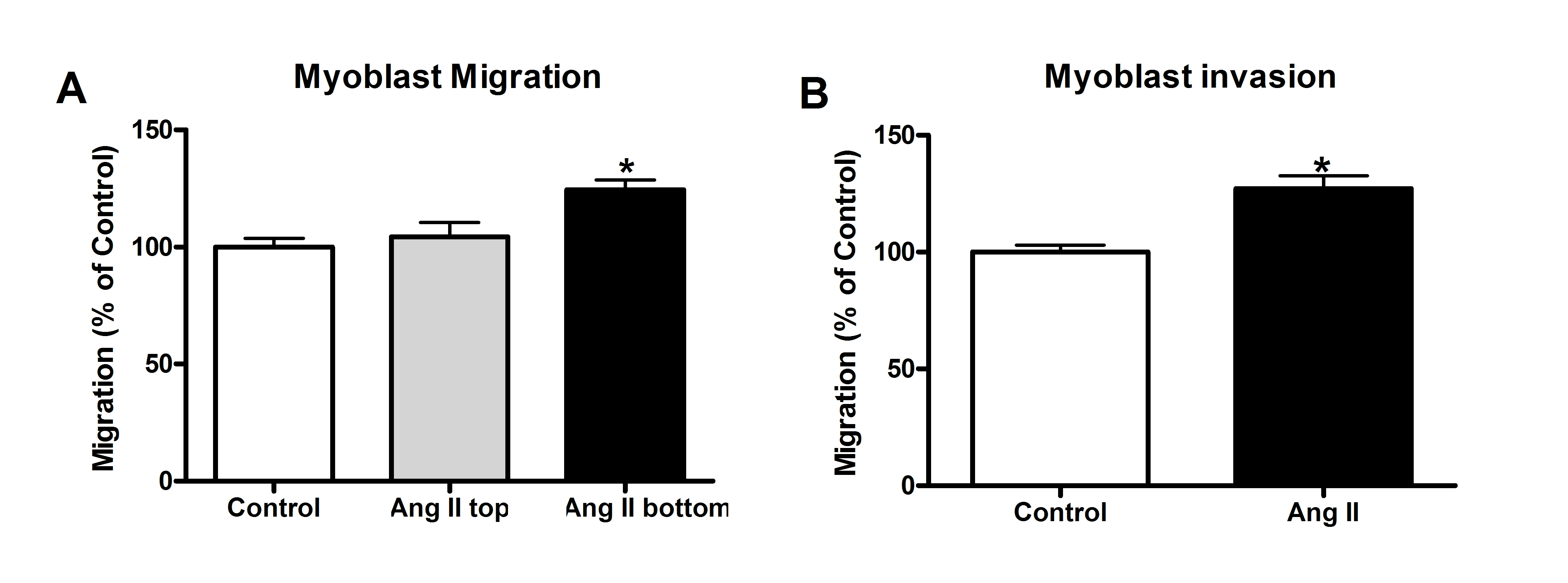

Supplement: Figure S4 — Ang II treatment increases myoblast chemotaxis and invasion. A) Analysis C2C12 myoblasts either directly treated (Ang II on top) or subjected to a concentration gradient (Ang II on bottom) of Ang II. B) Analysis of the capacity of control and Ang II treated C2C12 cells to invade gelatin coated transwells (n = 6 per group). Data are presented as mean ± s.e.m *indicates a significant difference (p<0.05) from control. (TIF) [file pone.0015212.s004.tif]

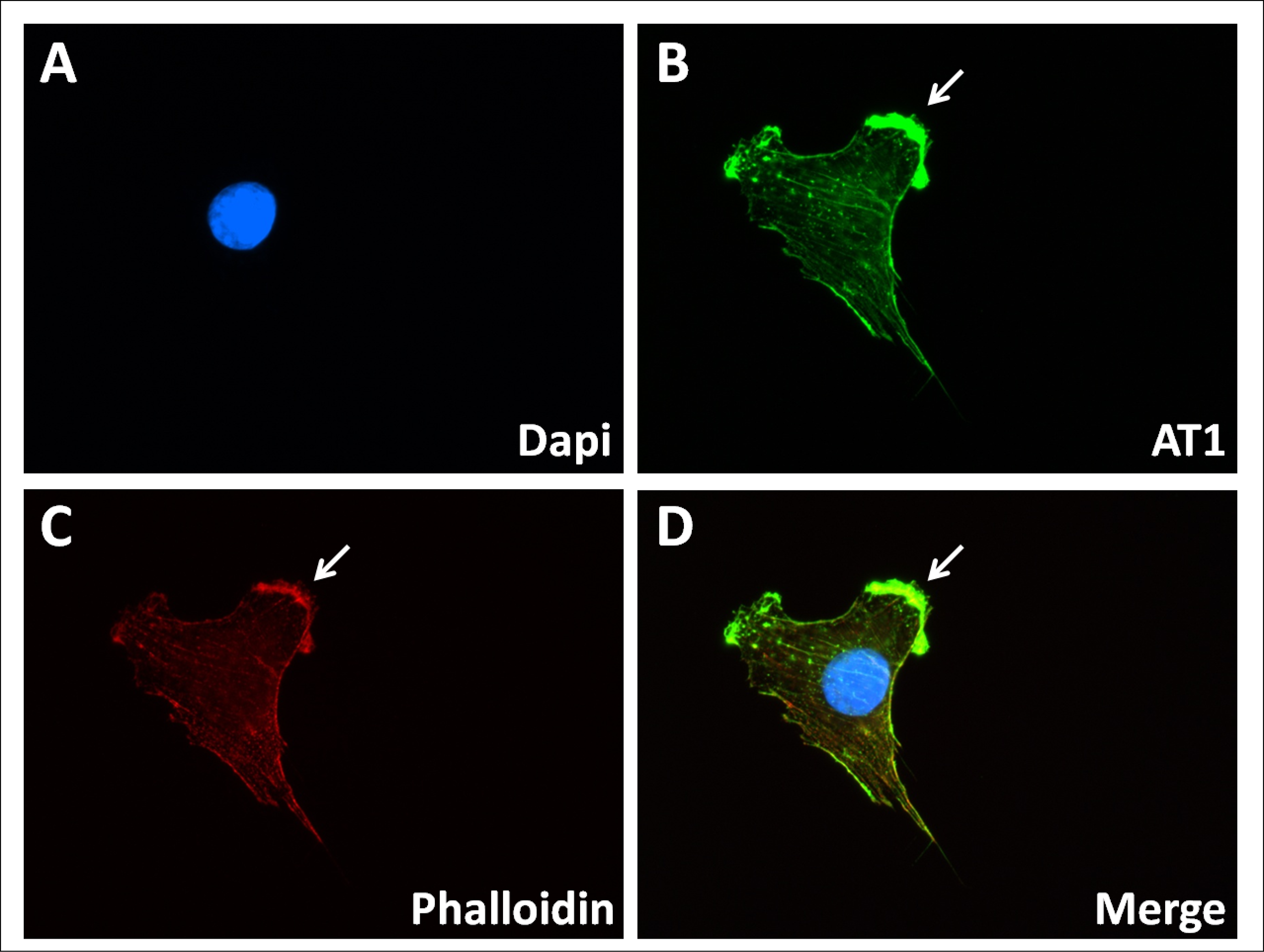

Supplement: Figure S5 — AT1 colocalizes with lamellipodial projections. IHC staining of (A) DAPI, (B) AT1, (C) phalloidin and (D) merge in C2C12 myoblasts (100x magnification). Arrows indicate colocalization of AT1 to lamellipodial projections of a polarized cell. (TIF) [file pone.0015212.s005.tif]

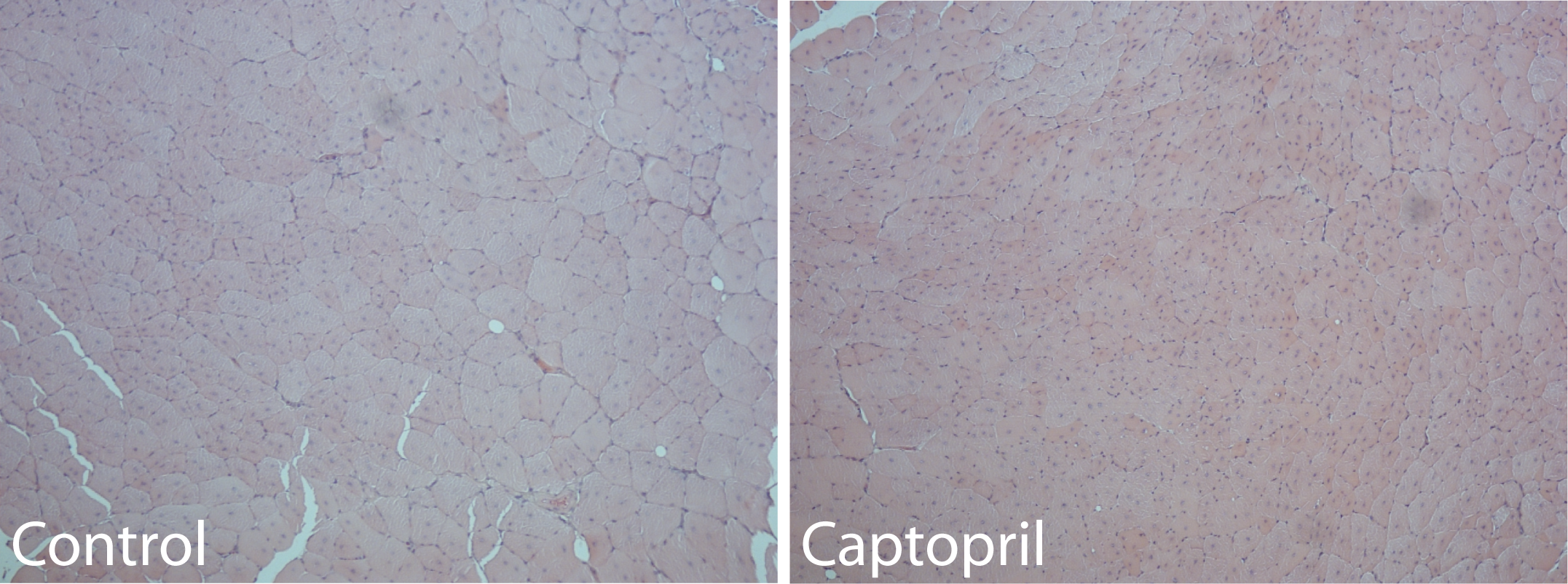

Supplement: Figure S6 — H&E stain of TA sections at 21d of regeneration. H&E stains at low magnification (10X) demonstrate that 1) all fibres contain central nuclei demonstrating homogeneity of injury, and 2) fibres from captopril treated animals were obviously smaller. (TIF) [file pone.0015212.s006.tif]

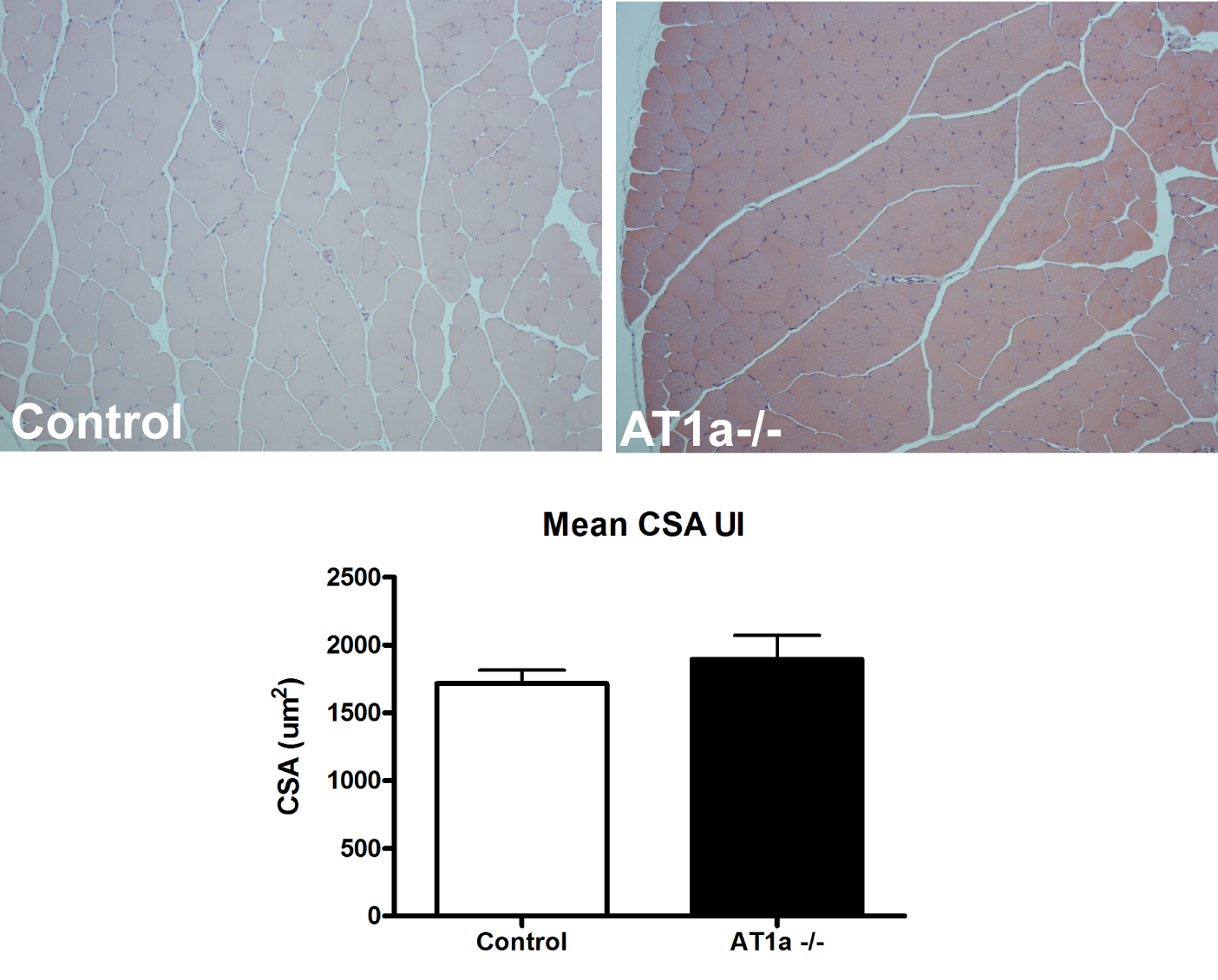

Supplement: Figure S7 — Comparison of AT1a−/− and control uninjured CSA. Representative sections of uninjured TA muscles from control and AT1a−/− mice demonstrate no difference in mean fibre CSA. (TIF) [file pone.0015212.s007.tif]

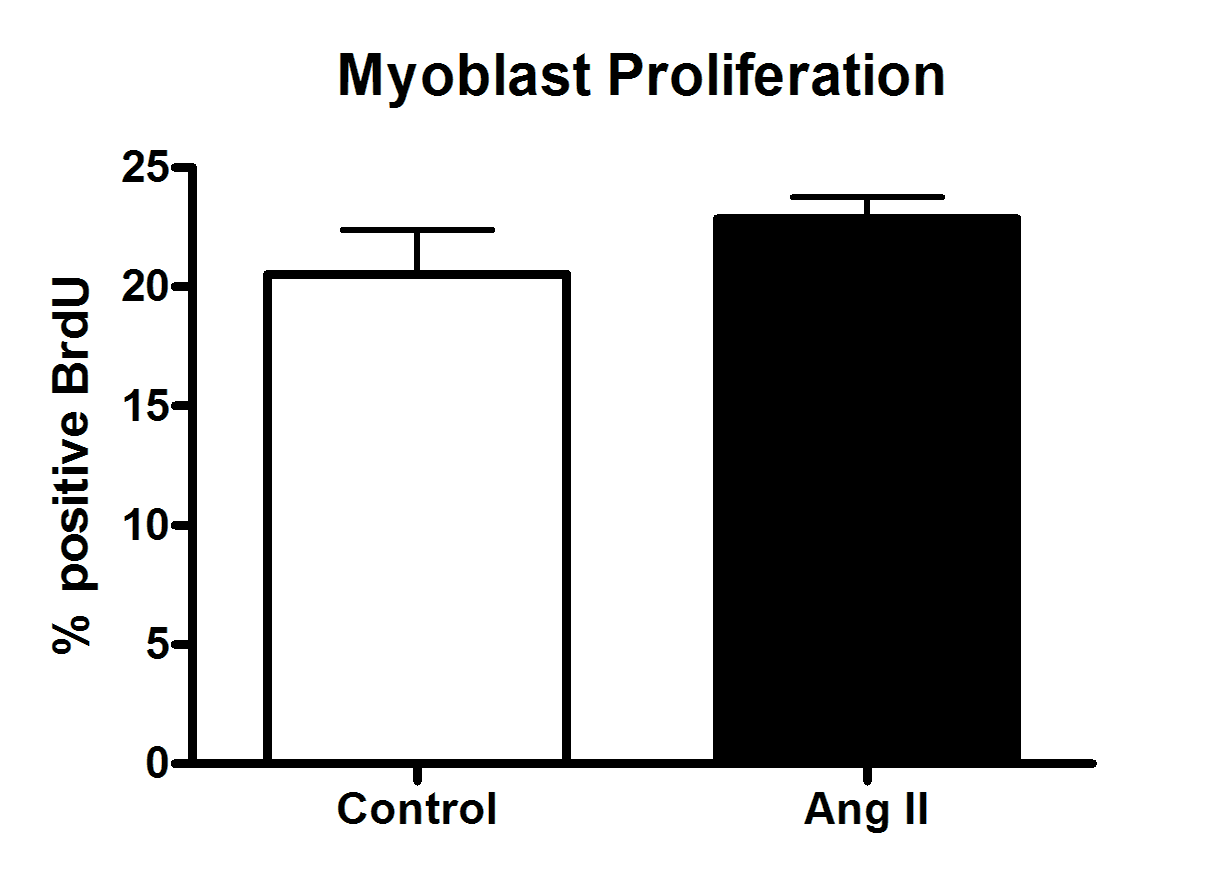

Supplement: Figure S8 — BrdU incorporation into freshly isolated satellite cells treated with Ang II. Additional evidence demonstrating that Ang II does not appear to influence cell proliferation. (TIF) [file pone.0015212.s008.tif]
